# Storage of Information and Its Implications for Human Development: A Dialectic Approach

**DOI:** 10.3389/fpsyg.2020.01715

**Published:** 2020-07-16

**Authors:** Gregorio Zlotnik, Aaron Vansintjan

**Affiliations:** ^1^Clinique de la Migraine de Montreal, Montreal, QC, Canada; ^2^Department of Film, Media, and Cultural Studies, Birkbeck, University of London, London, United Kingdom

**Keywords:** storage of information, consciousness, dialectics, cognition, extended cognition, human development

## Abstract

How has the storage of information shaped human cognition? We bring together current advances in cognitive science, the neurobiology of memory, and archeology to explore how storage of information affects consciousness. These fields strongly suggest that the increase in storage of information in the environment – which we call exosomatic storage of information – may have led to changes in human consciousness and human neurophysiology over time. To bring these findings together conceptually, we develop what we call a dialectical model of the relationship between storage of information and the development of human consciousness. Using the system of dialectic philosophy, we put forward that (a) environmental changes, consciousness, and neurophysiology develop together, (b) these changes were irreversible, (c) quantitative increases in exosomatic storage of information may have led to qualitative changes in human consciousness and neurophysiology, (d) these changes in turn affected how we see ourselves. Indeed, our capacity to store information exosomatically distinguishes us from other animals, and may be a key attribute of our self-awareness and therefore self-consciousness. Because metaphors are central to human thought and can help structure scientific inquiry, we illustrate our model using a metaphor of drops of silver on the back of a glass, eventually making a mirror – where successive quantitative change leads to an irreversible qualitative development in human consciousness. The dialectic model can offer new insights into the co-evolution of material culture and human beings through its broader philosophical foundations and explanatory power.

## Introduction

In Plato’s *Phaedrus*, Socrates tells the story of Thoth, who brings a wealth of inventions to the city of Thebes. One of these is the art of writing. Writing, he says, will make Egyptians wiser, because they will have better capacity to remember information. Amun, God of Thebes, had a different opinion: “this discovery of yours will create forgetfulness in the learners’ souls because they will not use their memories; they will trust to the external written characters and not remember of themselves” (Plato, 1892, p. 274d–275c). In Plato’s retelling of this myth, the god Amun seems to be aware that writing would change the way his people will think – they would rely on external storage of information rather than their own capacity to remember. And so he is cautious about letting Egyptians use Thoth’s invention.

The growth of information technology starting in the 1960s, and its explosion in the late 1990s through the development of the Internet, has allowed us to store information in ways that Thoth could have never dreamt of. We exchange photos with family and friends to share our daily experiences. We can record ourselves talking, let a program transcribe it, and save it to come back to later. Incredibly, scientists have succeeded in turning a DNA strand into a readable computer chip; fully blurring the line between organic and digital information storage (Church et al., 2012).

Though our situation today is much different from that of the ancient Egyptians, the myth of Thoth tells us that such questions about the effects of storage of information on our cognition are not new and have perplexed thinkers for millennia. We have been storing information externally by means of pen and paper, slates, and hieroglyphs for at least 8,000 years. The use of art, petroglyphs, cultural artifacts, traded goods, and specialized tools has been documented starting 300,000 years ago. How has the immense proliferation of information storage outside the brain affected the development of consciousness? Could it have really changed the way we think, as Amun feared?

To approach this question, we need to engage with four prominent fields: within cognitive science, the study of consciousness and the study of extended cognition; the neurobiology of memory (including epigenetics); and archeology. For one, studies of consciousness are coming closer to understanding awareness and self-awareness – and how we integrate various experiences into cognition over time (Ohta, 2005; Kandel, 2007; Graziano, 2013). More precisely, the development of Attention Schema Theory (AST) and similar theories of the mind offer a compelling model for integrating the role of environmental and social factors in structuring awareness and, therefore, consciousness (Graziano, 2013). Likewise, advances in the neurophysiology of memory have connected basic building blocks of information storage in neural networks to the creation of complex memories (Kandel, 2007). The field of epigenetics is highlighting that inheritance is not just a matter of DNA, but may also be experiential and tied to environmental factors (Allis and Jenuwein, 2016). Further, growing interest in extended and embodied cognition has led to an improved understanding in the role of the body, and the environment, in shaping consciousness. Extended cognition theories posit that external storage of information can become incorporated into cognition, therefore changing the way we think – and our neurophysiology (Clark, 2008a). Finally, archeology, and cognitive archeology in particular, points to the importance of material culture in the development of human cognition, as well as its interactions with neurophysiology. It turns out that Amun may have been justified in his fear that the availability of external storage of information would change his subjects irrevocably.

Yet, despite these advances, there is still no clear explanation for how, and in what way, the storage of information in the environment may have changed human consciousness and, thereby, our neurophysiology. That is, while current advances in cognitive theory and the study of memory offer an integrated approach to consciousness, its social and biological factors, and keys to its initial evolution, there is room to better understand how external factors such as the development of information storage may indeed alter human neurophysiology for good.

In this paper, we bring together current advances in cognitive science, the neurobiology of memory, and archeology to explore how storage of information affects consciousness. Summarizing the existing literature, we argue that the increase in storage of information may have led to qualitative shift in human consciousness and, therefore, human neurophysiology. To bring these fields together conceptually, we propose what we call a dialectical model of the relationship between storage of information and the development of human consciousness. Using the system of dialectic philosophy, we offer a framework to link the new science of mind and archeological findings. We put forward that (a) environmental changes, consciousness, and neurophysiology develop together, (b) these changes were irreversible, (c) quantitative increases in exosomatic storage of information may have led to qualitative changes in human consciousness and neurophysiology, (d) these changes in turn affected how we see ourselves. Indeed, our capacity to store information exosomatically distinguishes us from other animals, and may be a key attribute of our self-awareness and therefore self-consciousness. Because metaphors are central to human thought and can help structure scientific inquiry, we illustrate our model using a metaphor of drops of silver on the back of a glass, eventually making a mirror – where successive quantitative change leads to an irreversible qualitative development in human consciousness. The dialectic model can offer new insights into the co-evolution of material culture and human beings through its broader philosophical foundations and explanatory power.

## Contemporary Advances in Science of Memory, Cognitive Science, and Archeology

In the following, we summarize findings in research on the role of storage of information in cognition and the effect it may have had on human development. In the process, we will clarify contentious terms such as cognition, memory, consciousness, and external storage of information.

### Consciousness and Mind

Before proceeding, it is important to be clear about how we understand key concepts we are using. Consciousness is a famously vague term that has a wide variety of meanings. Following the work of other cognitive theorists, we conceptualize consciousness as a structure that has the capacity to receive, elaborate on, synthesize, and act upon information, including environmental awareness, alertness, and self-awareness. It is:

[A]n awareness of self, an awareness of being aware. Consciousness thus refers to our ability not simply to experience pleasure and pain but to attend to and reflect on our experiences, and to do so in the context of our immediate lives and our life history. (Kandel, 2007, p. 357).

Thus consciousness also involves an episodic memory, biographical memory, and mental time travel (Donald, 1995; Focquaert and Platek, 2007; Gärdenfors, 2007). The current and most accepted definitions of *mind* include a number of phenomena such as sensory perception, memory, consciousness, reasoning, control of emotions, and, possibly, determining action – an even broader (and more vague) term than consciousness! Neurosurgeon Tomio Ohta (2005) has proposed that consciousness and mind are separate processes, with memory and language acting as liaison officers between them. From this perspective, consciousness is distinct in that it is a structure that receives information provided by the senses and supervises the process by which the mind and brain elaborate this information (Goldberg, 2002). This indicates a narrower use of the term *mind* – thus we avoid using it wherever possible, instead using terms like “consciousness,” “cognition,” and “mind-brain.”

Recently, AST, as proposed by Graziano (2013, 2019) and Graziano and Webb, 2014), has offered a coherent and promising model that explains how consciousness functions as an attention regulator and data-handling process. In this theory, attention and an attention schema are different processes, in the same way that the body and the body schema – which is a representation of the body in our mind-brain – may be linked but are not the same. It is argued that attention and the attention schema co-evolved over roughly 500 million years. Apart from applications in machine learning and AI, the theory also offers some clues as to how cultural and environmental phenomena became integrated with, and eventually modified, cognition (Graziano, 2013; Frödin, 2017). In particular, it offers an inter-disciplinary model for how social and natural events, as well as internal cognitive processes, may act in concert and together modify consciousness. This gives us an explanation for the feedback between cultural and social norms and neural processes, which in turn shape cognition and learned behavior – thus helping to resolve dualistic notions of “nature vs. nurture.”

There are a few gaps in this theory worth pointing out here. First, underlying AST is a static representation of the mind-brain, where an “architecture” structures “general external parameters for the types of connections, cognitive styles, and forms of sociality that humans can activate and draw on” (Frödin, 2017, p. 15). Mind-brain “architecture” is conceived of as a condition of evolution while society may change it over time, however, there is little explanation of the dynamics of these changes. Second, external and internal phenomena are opposed dualistically – an issue which we further explore in our discussion on extended cognition. Third, and relatedly, though there is room in the theory for feedback processes between learned behavior and neurophysiology, as well as the role of neuroplasticity in shaping cognition, there is little engagement with the role of storage of information and the possibility that it may in fact transform human cognition *permanently* – i.e., the unique role that our ability to store massive amounts of information in our environment may have had in the development of modern consciousness. Indeed, the theory glosses over an important period in human development, when we may have evolved from anatomically modern humans 300,000 years ago, to the present (Graziano, 2013, 2019), and the documented changes in human neurophysiology that have happened since. As we argue below, understanding the role this period has had in human development – and therefore its effects on consciousness – requires being more clear on how storage of information, neurophysiology, and consciousness interact.

### Memory and Neurobiology

*Memory*, in contrast to consciousness, is more concisely defined: the encoding, storing, and retrieving of information (Squire, 2009). The science of memory, and our understanding of memory storage in living organisms, has developed considerably. By the end of 19th century, it was proposed that the storage of memories in animals does not happen through the creation of new neurons, but rather through a “strengthening” of connections between neurons (Ramón y Cajal, 1894). Famously, research by Bliss and Lømo suggested that the formation of memories may largely involve coded modification to the strength of chemical synapses (Bliss and Lømo, 1973) – what is called long-term potentiation (LTP).

Current advances in the study of memory are further outlining the role of environmental factors in the storage of memory. The discovery of epigenetics raised the question of how memory may get stored, not just through experience, but also transgenerationally (Allis and Jenuwein, 2016). Advances in the field suggest that, on a molecular level, diet and other environmental factors have an effect on offspring metabolism and possibly lead to transgenerational inheritance (Carone et al., 2010; Öst et al., 2014). Incredibly, recent findings even suggest that behavior, memory, and learning could also be passed down transgenerationally (Allis and Jenuwein, 2016; Posner et al., 2019). This raises the possibility that the accumulation of memory over time, including environmental factors, may in turn alter the human cognitive experience irreversibly and, by extension, human neurophysiology.

All in all, we no longer understand memory as a more internal, personal, subjective, even ephemeral experience. Rather it is physiological, biological, chemical, and can be studied through scientific inquiry (Kandel, 2007). Given these implications, some may argue for holding on to the idea of memory as an internal process, a subjective experience that requires consciousness. Instead, we should now accept an extended definition of memory: the storage and retrieval of information (Zlotnik and Vansintjan, 2019).

### Extended Cognition

It’s not just our concept of memory that is expanding. We are coming to terms with the idea that human cognition *in general* may extend beyond the brain. Proponents of E4 cognition theory argue that mental processes are embodied (e.g., in the body), embedded (within an environmental context), enacted (namely, dependent on an organism’s activity), and extended (relying on information storage beyond the brain) (Clark and Chalmers, 1998; Clark, 2008a; Menary, 2010; Rowlands, 2010; Favela and Chemero, 2016; Briglia et al., 2018). The last attribute of the E4 cognition thesis is especially relevant today, since we are in an era where massive amounts of information are encoded within and permeate our daily experience. Technological advances emphasize how we are already in great part cyborgs: prosthetic limbs, smartphones, GPS devices – these are techniques that change the way we interact with the world and have become integrated into our motor reflexes. These are each forms of “extended memory” that these authors argue have become, and will increasingly become, incorporated into cognition.

Note that in this paper we prefer to use “exosomatic” over “external.” Exosomatic storage of information also has internal properties, and using the term “external” is therefore a misnomer. We also caution against confusing information with the object itself. We view information as the “software” while the medium is the “hardware”; they share a material connection but the former requires socially learned experiences to be legible. For example, the development of walls, roofs, and even furniture may initially have been discovered through trial and error, but their further refinement requires blueprints and passing on of information on construction methods, as well as sharing information on how to use them. Each of these media have their own dynamic and rules in terms of how they may enable the storage of information. We also highlight that there is a unique difference between exosomatic storage of information, which can be intentionally stored and retrieved, and other artifacts which we interact with regularly. For example, a utensil is more of an extension of the body (embodied cognition) and does not exist solely for the purpose of being stored and retrieved – thus highlighting the importance of intentionality for exosomatic storage of information (Searle, 1983). Further, we prefer to use the term “storage of information” instead of “memory” – the latter of which often suggests a purely “internal” process. For us, storage of information may occur both within the brain and the environment – though these realms should not be dualistically opposed and what we call the “mind” may include both. This, however, means that any “external” (i.e., exosomatic) storage of information by humans must necessarily also correspond to physiological “internal” (i.e., endosomatic) activity as it requires acquisition and information classification within the brain.

So how could exosomatic storage of information also be physiological, and how does it, in turn, affect our mental states and consciousness? In other words, how is exosomatic storage of information *materially embedded* in our brain? To account for this, Clark (2008a, p. 37) proposes that the mind-brain “incorporates” memories. This is one productive way of thinking about the process. Symbiotic incorporation is a commonly occurring process in nature. For example, there is evidence that early cells incorporated mitochondria into their own cell structure (Thrash et al., 2011; Ferla et al., 2013). External processes can become internal, changing both. Likewise, the development of exosomatic information storage is incorporated in the brain, necessarily changing physiological processes and thereby potentially changing consciousness. This is in contrast to an understanding of the brain as physiologically static, where, despite exosomatic changes, the brain and consciousness do not themselves change over time. Others have pointed to the relevance of niche construction models, which focuses on the role of the environment in the development of cognition. As Kim Sterelny points out, niche construction, in particular the co-evolution of exosomatic storage of information, child development, environmental changes, may better explain how the mind came to be supported by material, exosomatic “scaffolding” and how this then became integrated into the mind-brain (Sterelny, 2010).

“The big picture idea depends on accepting nativist psychology’s contention that our everyday cognitive skills depend on the mastery of large and often subtle bodies of information, while rejecting the idea that the core of these informational resources is innate, preinstalled by evolution.”

However, while the outline of a framework for understanding how exosomatic storage of information may alter neurophysiology is there, there is room for more exploration of, first, the more detailed dynamics of how this may occur; second, how this may interact with epigenetic processes; and, third, the role of the development of storage of information for the development of human cognition over time.

### Storage of Information and History of Human Development: Archeological Evidence

How has our capacity to store information changed throughout human history? Asking this question leads us to immediately follow up with another question: when did we first start storing information outside of the body? Evidence for the storage of information by humans in the environment exists for up to 300,000 years ago, through the use of beads, ochre, and other sporadic cultural objects. Artifacts such as specialized stone tools, cave paintings, petroglyphs, ceramics, figurines, eyed needles, textiles, and human settlements began to become more ubiquitous in the archeological record around 50,000 years ago (Barham, 2013; Gamble, 2013). This period, especially from data gathered in the European and Middle Eastern archeological records, has been called the “Upper Paleolithic.” Some have even argued that, because of this unique development, behaviorally “modern” humans emerged during this period (Klein, 2009). However, this classification has been questioned in the literature, especially considering newer archeological findings in Africa (McBrearty and Brooks, 2000; Galway-Witham et al., 2019). The “explosion” of artifacts may indeed be due to a more highly concentrated population and other factors (Galway-Witham et al., 2019). It is likely that modern human behavior originated in Africa as part of an “older, more gradual behavioral package” (Galway-Witham et al., 2019, p. 363). What is clear, however, is that the archeological record points to the development of artifacts throughout the last 300,000 years which continued to accelerate with human expansion into Eurasia, Asia, the Pacific, and, eventually, the Americas (McBrearty and Brooks, 2000; Stringer, 2011; Gamble, 2013). It is important to further specify, however, that the storage of information was not intentionally *invented* over time, rather, humans increasingly *discovered* more ways to store information, which led to changes in how people interacted with each other and the environment.

The spread and diversity of artifacts can also be seen as an indicator for changes in the human capacity to store information outside of the body. It has been argued that the increased presence of artifacts represented a cultural evolutionary “tipping point” in the storage of information (Bentley and O’Brien, 2012; Valverde, 2016). This is proposed to be a catalyst in human development, similar in importance to the development of language. There are several proposals for why exosomatic storage of information is so important for human development. For one, exosomatic storage of information is key for cultural transmission, next to sharing of information about distant or metaphysical entities and the institutionalization of social norms (Heyes, 1993, p. 1006). There is little research that identifies any of these activities in non-human animals; these are seen to be unique to the human experience (Heyes, 1993; Sterelny, 2010; Galway-Witham et al., 2019). Further, the emergence of visual art, dance, music, and written symbols is also argued to have had a unique impact on human ecology, culture, and neurophsyiology (Hoffecker, 2013). Finally, it is suggested that there was an interplay between technological development and cultural behavior and physiology. These were possibly self-reinforcing, since these developments could help secure the energy needs of a larger brain and a larger brain could also lead to more diversity in and capacity for cultural and technological development (Sterelny, 2010; Antón and Snodgrass, 2012; Galway-Witham et al., 2019). Indeed, there is evidence that modern behavior stabilized 150,000 years ago, around the time that the parietal lobe also reached its current dimensions (Schlebusch et al., 2017; Bruner, 2018; Neubauer et al., 2018; Davies, 2019). Thus, the development of storage of information was not simply a result of encephalisation (growth of brain size), but required the evolution of different social systems, “such as the extension of scale of ranging areas and interaction networks, the appearance of social norms relating to information and its transfer, or the use of material cultural symbols as information stores” (Steele and Shennan, 1996, p. 26). In summary, cultural transmission, a defining feature of modern human behavior, is contingent on the ability to store information exosomatically. Further, this ability to store and retrieve information in the environment is unique to humans, and likely led to an interplay between cultural evolution and human (neuro)physiology.

What can explain this relationship between exosomatic storage of information and human development? Within archeology and evolutionary anthropology, these dynamics are often discussed as coevolution or, alternatively, material agency. Coevolution refers to the biological process of reciprocal evolution between species. Biocultural coevolution refers to how culture may also have a coevolutionary effect, where the development of culture may affect the development of a species and/or its physiology and genetic makeup (Richerson and Boyd, 2005). Culture is seen as a dynamic evolutionary property that impacts the evolution of a population as a whole. It has been proposed that cultural evolution was enabled when humans developed the cognitive ability to recognize others as agents, as a kind of “ratchet effect” (Tomasello, 1999). A ratchet is a mechanism that consists of a wheel or cog with teeth, which are then engaged by another cog. The ratchet can only be turned forward, not backward. The whole may move while the center stays in place. In the context of cultural coevolution, the metaphor is intended to illustrate how cognitive abilities evolved to produce cultural behavior, which then led to an irreversible process where culture in turn affected human evolution. A similar proposal is the Baldwin effect, which puts forward that new learned behaviors can shape a species’ survival and therefore their evolution (Baldwin, 1896). Material agency, on the other hand, refers to the centrality of non-human objects in driving human evolution, development, and cultural change (Knappett and Malafouris, 2008). There are overlaps here with extended cognition and embodied cognition theory, as material agency is seen as an important requirement for cognition to be an interaction between the environment and the mind-brain (see, e.g., Clark, 2008b; Ingold, 2008). Yet, there is room for more exploration of the dynamics by which storage of information may have interacted with human neurophysiology as it started to be developed by humans and began to shape our environment – especially in terms of the neurobiology of memory, extended cognition, and the newer conceptions of consciousness such as AST (Malafouris, 2010a,b, 2015).

### Summary and Implications of Advances in the Fields of Cognitive Sciences, the Study of Memory, and Archeology

These new directions in the study of memory, human origins, and consciousness are incredible, even somewhat bewildering. While we don’t have to accept all of their conclusions and, indeed, much of this research is still a work in progress, we can draw out a few broader implications. First, as emphasized by AST, a dualistic approach to consciousness – which separates representational, conscious experiences from organic, physiological processes – must be rejected. However, there is more to explore in terms of the role of storage of information and how it may transform the architecture of the brain – a term which, itself, implies rigidity and permanence. Similarly, while E4 cognition theories offer a broader understanding of the mind-brain, there is little precise explanation of how exosomatic storage of information may itself be incorporated in the mind-brain, and how this may have happened over time. Second, it is clear that memory storage and retrieval is an organic, physiological process, that is, it is an emergent property of the mind-brain. Further, advances in the study of epigenetics suggest that memory may be passed down along generations and that genetics alone are not the sole marker of the evolutionary process. Third, memory is not reducible to representation or conscious experience alone – it extends to the retrieval and storage of information more generally. That is, memory is certainly a key component of consciousness, and may shape it in new and unique ways. But it itself does not require consciousness. For this reason it may be preferable to extend the definition of memory to include the storage of information more generally (including endosomatic and exosomatic storage of information). Fourth, developments in archeology and the study of human origins further add to the picture and emphasize that exosomatic storage of information did have an important role in human development. Further, it is clear that storing information in the environment is unique to humans and therefore extended cognition is as well. Yet, concepts such as cultural coevolution and material agency do not sufficiently discuss the role of storage of information in these dynamics, and remain limited to arguing that interactions are plastic, rather than more precisely defining the way by which those interactions take place and the developmental effects they may have had. It is also unclear what kind of interaction the development of exosomatic storage of information may have had with human neurophysiology, and the impact that this may have had on human consciousness more generally.

In the following, we do not seek to resolve or answer these questions. It is clear that more research will be necessary in exploring the role of storage of information in human development over time. Indeed, some questions may never be conclusively answered. In the following, however, we wish to present a framework that can contribute in the *framing* of these questions through offering an especially relevant philosophical foundation. We propose that dialectical philosophy, in particular G. W. F. Hegel’s dialectic method, can help us conceive how exosomatic storage of information may lead to irreversible changes in endosomatic, cognitive processes.

## The Contributions of a Dialectical Model

In the following, we outline the dialectical model as it pertains to the role of storage of information in human development. Here we rely largely on Hegelian dialectics (Hegel, 1807/1979, 1812/2014). Since Hegel developed his thought, others have also advanced dialectics, which we cannot explore fully here^1^. We explain Hegel’s dialectics through four main points. However, suffice it to say that these principles continue throughout contemporary dialectic thought.

The first principle is to go beyond dualistic thinking. One of the difficulties of understanding how the storage of memory occurs within the brain/mind is that we often tend to separate the physical property (e.g., chemical encoding in neural networks) with the effect (a subjective, personal memory). Dualistic thought is considered a common problem within the history of philosophy. Philosophers, such as Plato, Descartes, and Kant sought to overcome this problem by stressing either the absolute reality of ideas or of matter. Each of these approaches were unsatisfactory because they retained dualism between ideas and matter in some form or other (see, e.g., Bhaskar, 1994). Hegel, in contrast, proposed that ideas and matter are opposites that require each other. It’s impossible to conceive of ideas without conceiving of matter; similarly it’s impossible to conceive of being without conceiving of non-being, or culture without nature, the unknowable without the knowable. Further, neither identities are static. Just as matter develops and changes over time, thought must change throughout time. Hegel sought to move from a dualistic conception of the world toward a dialectic conception, where thought and matter, ideas and nature, are seen as co-constitutive. This is important for our own discussion, because our problem, as stated above, is that we cannot accept a dualistic approach to the role of advances in information storage in development of human cognition.

The second principle is what can be called “sublation,” that is, an interaction between a thesis and antithesis, which necessarily ends in a new synthesis, which is irreversible and conserves some aspects of both. A classic example is that of “being” and “non-being.” To think of being requires thinking of its antithesis, “non-being.” The second is thus sublated into the first, in what is called “becoming,” which carries both what it *is*, and what it *is not yet*. This principle is also applicable to historical developments. One example is capitalism and socialism. The development of capitalism, based on private property and universal exchange of money, led to the articulation of its antithesis, where all is held in common and monetary value is obsolete. Social democracy is the sublation of the two. Another classic example is the master-slave dialectic, as proposed by Hegel. Two men confront each other. One becomes the master, and the other the slave. The slave makes things for the master, learns, develops skills, develops creativity, and sees himself in the products of his labor, thus becoming self-conscious. The master, however, is dependent on the slave’s production, and is eventually constrained by his slave’s labor. This myth describes how two entities change each other through their interaction, which in turn changes their environment, which in turn changes them once again.

In sublation, fixed ideas become unstable, leading to the opposite idea, leading to a positive synthesis. Sublation also occurs in nature. Cells absorb mitochondria into their walls, creating a eukaryote; electrons and positrons cancel each other out, creating gamma ray photons. Importantly, sublation is a dynamic, organic process; everything is in relation with other things. For Hegel, all of nature displays this kind of tension. This distinguishes his work from mechanistic approaches, which require the introduction of an external impulse to explain cause and effect. By “mechanistic,” we mean an understanding of change that assumes predictable, determinable relationships between distinct and independent parts, the summary of which can explain any event – this is otherwise called determinism. Mechanistic thinking is useful in many contexts, for example, in engineering. However, it is insufficient in ecology, social science, and the study of history. A dialectic approach does not assume predictability, but, rather, is a way of analyzing historical developments after the fact. From a dialectic perspective, humans both shape and are constricted by social, economic, environmental, and technological processes. In its non-mechanistic approach, dialectic thought is similar to the principles of coevolution and material agency, in that it understands that two or more processes may initially have been distinct, but eventually each alters the other. However, it differs from these principles in that it is attached to a body of thought which seeks to integrate an understanding of human cognition with how we understand and perceive the world and how that, in turn, is shaped by historical, cultural, and environmental developments.

This non-mechanistic aspect of dialectic philosophy can be better understood if we consider Hegel’s distinction between quantitative and qualitative change, our third concept. Quantity is the amount of a thing, where the amount does not change the thing in itself. Quality is the character of a thing. Quantitative change occurs progressively, while qualitative change indicates a change of state. For example, growing one, two, or three trees doesn’t make a forest. A forest is created when a certain critical mass of trees grow together, creating a distinct ecosystem composed of many trees. Another example is that of a raindrop hanging from a leaf, which Niels Bohr used to explain the process of nuclear fission. Tension between the water surface and gravity allows the drop to hold together. However, as the quantity of water collected within the drop accumulates, it reaches a critical limit and, eventually, the tension breaks, and the raindrop falls. A final example can be taken from land use regulation law. In American jurisprudence, land use regulation is permitted on the municipal level – but what is “too much” regulation? As the legal scholar and Hegel expert David Gray Carlson notes, “regulation’s quantitative burden can be gradually increased with no qualitative change, but there comes a sudden moment when quantitative change is so great that a qualitative change is effected” (Carlson, 2002, p. 16). However, this moment is difficult to predict, “neither the Supreme Court nor its innumerable interpreters can say in advance what constitutes too much regulation, just as we can never specify the exact hair that, if extracted, makes a man bald” (Carlson, 2002, p. 17). Also importantly, this kind of theory of change is non-linear. It is non-linear because change appears as chaotic and difficult to predict or define, but nevertheless it is not random. In this way, the distinction between quantitative and qualitative change further underlines the point that mechanistic explanations cannot ultimately do without qualitative description of change.

A fourth concept from Hegel is the notion of progress or historical development. According to Hegel, all historical processes create a new reality. At every single moment in time, there is always movement, opposition, and resolution. Though Hegel didn’t use the word *Zeitgeist* – he preferred to use *Geist der Zeiten* – it is often used in connection to his work. However, the concept should be used with caution, it is not what it is often caricatured to be, as a “fashion” of the time, or as some kind of mystical force determining history and everyone’s actions. It just means that ideas and ways of thinking are products of their own time and may not occur in the same way in other contexts and periods. We are always progressing, but it is not clear to what. But what is clear is that we are moving forward and the past is left behind. This idea of progress was similar to that of Charles Darwin, who proposed that evolution tends toward improvement and adaptation – as he noted, “Progress has been much more general than retrogression” (Darwin, 1871, p. 177). Further, Hegel is often criticized of being too teleological – that there is a steady march of progress, at the end of which we will approximate God. However, use of the words “progress” or “development” should not imply that there is an end in sight – this “end” is constantly receding, and this is the tension within the dialectic process itself. Like a ratchet, historical developments are permanent. But unlike a ratchet, they occur organically and non-mechanistically.

## Implications of a Dialectic Approach to Storage of Information

We have summarized four key points of dialectic philosophy. These are: the move beyond dualism, sublation, quantitative vs. qualitative change, and progress/development. We put forward that a dialectic approach offers a unique framework to think about the dynamics between exosomatic storage of information, consciousness, and human neurophysiology – which improve on the models of material agency and coevolution.

First, and more obviously, a dialectic approach supports current frameworks, such as AST and E4 cognition, which seek to move away from a dualistic approach to the interaction between consciousness and the environment. Changes in the environment lead to changes in perception and embodied cognition, which in turn leads to changes in human neurophysiology. In this way a dialectic approach is also in line with concepts such as material agency and coevolution, which represent less mechanistic, and more organic, ways to understand the interaction between humans, culture, and their environment. It also underlines the possibility that individual experiences may be inherited and passed on transgenerationally, which would align with new developments in evolutionary, biological, and ecological sciences, as well as in epigenetics (Huneman and Walsh, 2017).

Second, a dialectic approach highlights how storing and retrieving information external to the body also would have physiological consequences – and that this would happen when cumulative quantitative change leads to qualitative changes. When the brain retrieves the stored information, it reacts, learns, and produces changes. These changes enhance (change) the brain. This a dialectical change. Then, if we agree that, eventually, any progressive increase in quantitative change will lead to a qualitative change in the system, we will need to consider that there is a theoretical point at which an increase in the external storage of memory would change the brain. It is here that a dialectic approach differs from concepts such as coevolution, as it proposes that change would not necessarily happen *gradually*, but rather *eventually*, through cumulative interactions. Indeed, this possibility of quantitative change in exosomatic storage of information leading to qualitative changes in human consciousness is certainly already appreciated (e.g., Bentley and O’Brien, 2012), but requires more exploration.

Third, Hegel’s dialectic philosophy also highlights the possible *irreversibility* of these changes. Here, we do not mean that these changes were *permanent* or *static*, but that, once introduced, we could no longer go back to a time before them. Terms like “architecture of the brain” and “we are hard-wired,” as often used in cognitive science and evolutionary psychology, suggest that the mind is static while society changes. However, as Hegel argued, consciousness is not fixed but dependent on the historical context. This is what philosophers – inspired by Hegel – have called the historicity of consciousness (Husserl, 1970). Indeed, as discussed, the study of epigenetics indicates the possibility that information may be passed on transgenerationally while DNA stays largely the same (Klosin et al., 2017; Posner et al., 2019). Ecological changes and diets – products of changes in human sociability – further impacted human cognition and may have contributed to the development of the more modern mind-brain (Galway-Witham et al., 2019). Fields of anthropology and archeology also support the notion that human consciousness may be subject to environmental, social, and historical changes (Rowlands, 2004; Gamble, 2007, p. 162; Galway-Witham et al., 2019). Over and beyond concepts like coevolution and material agency, however, a dialectic approach emphasizes that one could not go back to a time before these changes: human consciousness has changed for good, and continues to change, as it continues to interact with its environments.

Fourth, and finally, we propose that a dialectic approach also points to the role that a change in exosomatic storage of information may have in our self-awareness. Consider the myth of the master-slave dialectic once again, where the relationship between the two ended up shaping each character’s idea of himself. Indeed, a dialectic approach would further underline how the development of consciousness due to our interactions with our surroundings would also shape how we see ourselves – and sets us apart from others in the animal kingdom. Cognitive theorists and archeologists also support this. Damasio (1999) argues that identity, what he calls an “autobiographical self” is an important component of consciousness, which is constituted by accumulated memories (and exosomatically stored information, we would add). He argues that humans have access to an extended consciousness, which other primates or animals do not. Neuroscientist and Nobel prize winner Kandel thus claimed that “We are who we are in great measure because of what we learn, and what we remember” (2006, p. 10). Gamble (2007, p. 118) adds that we are able to develop this kind of extended consciousness “because of our ability, using metaphor, to construct concept from [accumulated experiences]. These experiences are not just linguistic in origin but related to objects and bodies that structure the performance of social life.” Thus, though it may be correct to say that consciousness is by and large static for most animals, our social and cultural life, paired with our ability to store information externally, means that we are in a unique position in the animal kingdom, where, as we transform our surroundings through our ability to store and retrieve information, we also transform the way we see ourselves. Indeed, these insights were taken up further by 20th century philosophers, who drew on his dialectic method to put forward that any society which limits education and the acquisition of knowledge, would also limit human freedom. Thus, a society that focuses only on the creation of employment or profit margins, rather than education and culture would also limit human development (Vygotsky, 1987; Bhaskar, 1993; Harré and Gillett, 1994).

To summarize: we suggest that exosomatic storage of information would have been crucial for the development of an autobiographical self, and therefore of brain/mind processes and consciousness more generally. This is because external storage of information has the unique character of extending our self into our environment. As more and more information is stored outside of our brain, our experience of the environment would change as something that increasingly reflects on ourselves. Though the ability to store information exosomatically is not unique to humans, the scale of our ability to do so, and our ability to incorporate them within our own identity and consciousness, is unmatched in the animal kingdom. A dialectic perspective highlights that a quantitative difference in our capacity to store information also, at a certain point, requires a qualitative shift in experience. Furthermore, this qualitative change, or development, is irreversible. Finally, it is organic and material, where a change in perception also implies a physiological change.

## Understanding the Contributions of a Dialectic Approach Through the Metaphor of a Mirror

Metaphors are central to human thought (Gamble, 2007) and can help structure scientific inquiry. Take one metaphor that has been used to describe human development, the ratchet effect, described above. This metaphor is used to explain cultural coevolution (Boesch and Tomasello, 1998, p. 602, cited in Gamble, 2007, p. 168). Humans develop certain technologies that improve their position, which are then not abandoned, but improved upon. However, as Gamble notes (Gamble, 2007, p. 168) this metaphor continues to take a mechanistic view of historical and human development. Change may be conceived as irreversible, but the methaphor also connotes linearity, two-dimensionality, and mechanistic, rather than organic, change.

We think another metaphor helps us better illustrate our point: *Drops of silver land on a plate of glass. As more drops of silver accumulate, you see yourself fully, as in a mirror.* Like the ratchet, the process is also irreversible. But in this case, change is not linear or mechanistic. It also underlines the relationship between a change in the environment and the development of self-awareness.

We use this metaphor as a way to imagine what it might be like to discover and invent new ways of storing information exosomatically. Though, initially, the process might seem sporadic or without a clear goal, eventually a pattern would emerge and a qualitative shift in experience would occur. Further, this process can’t be reversed: once we start storing information in our environment – or painting the glass – it stays there and affects future generations. The increase in silver represents increase in knowledge, which allows us to see ourselves better. Thus the autobiographical self is born.

We do not intend this metaphor to imply that once the mirror is fully painted, no more change or development is possible. This would, indeed, run counter to a dialectical conception of change. Rather, it is meant to underline how change in the quantity of exosomatic storage of information may lead to qualitative change in the experience of consciousness. Indeed, as we increasingly place parts of ourselves in our surroundings, those surroundings may then reflect back on our own consciousness, changing us and how we see ourselves.

## Conclusion

There has been no detectable change in human DNA, brought about by biological evolution, in the ten thousand years of recorded history. But the amount of knowledge handed on from generation to generation has grown enormously. Some people would use the term, evolution, only for the internally transmitted genetic material, and would object to it being applied to information handed down externally. But I think that is too narrow a view. We are more than just our genes. We may be no stronger, or inherently more intelligent, than our cave man ancestors. But what distinguishes us from them, is the knowledge that we have accumulated over the last ten thousand years, and particularly, over the last three hundred. I think it is legitimate to take a broader view, and include externally transmitted information, as well as DNA, in the evolution of the human race. (Hawking, 1996).

In one lecture, “Life in the universe,” esteemed physicist Stephen Hawking noted that the development of exosomatic information storage should be considered a form of evolution. Like the Egyptian god Amun, Hawking believes that writing fundamentally changed people. In this paper, we take a broader understanding of exosomatic information storage and discuss how current advances in the study of memory, cognition, and archeology point to the important role that storage of information has in shaping cognition. However, unlike Hawking, we are less sure that we can say anything definitive about the definition of human nature, since we take a more dialectic and organic, and less teleological and universalist, approach to historical development.

What is clear, however, is that, since Hawking advanced his theory several decades ago, disparate fields have converged and there is now more evidence to explain how exosomatic storage of information has had a significant impact on human development, causing irreversible change, to the degree that it has likely affected our cognition and neurophysiology. AST and E4 cognition theories offer a view of human cognition that integrates our surroundings, culture, and neurophysiology into a coherent model. The study of memory and neurobiology highlights the role that environmental factors may have in the storage and retrieval of memory, and suggests that memory may be passed down transgenerationally. Archeology has highlighted that storage of information has had an important role in human development, especially in the last 150,000 years.

However, while these fields continue to advance, there is still need for reflection on the role of storage of information in human evolution. A dialectic perspective, as developed by Hegel two centuries ago, gives us a framework by which we can understand how a quantitative shift in the storage of information may have led to a qualitative change in human consciousness. That the exosomatic storage of information may shape our consciousness and neurophysiology does not imply a hierarchy between different material cultures, rather, it further underlines the plasticity, diversity, and potential of the human being, and its distinctiveness from other animals. A dialectic approach also helps us conceptualize how exosomatically stored information may be incorporated physiologically in the mind-brain and how this may then be, in turn, passed down over generations. Rather than a mechanistic metaphor of a ratchet, we suggest the imagery of a plate of glass, slowly covered, seemingly at random, with drops of silver. This metaphor, we think, highlights how a process that may have initially been external to us can change us internally. It also highlights the role of the “autobiographical self” in human consciousness, and points to the possibility that exosomatic storage of information has been an important agent in the development of consciousness over time. Metaphors, we believe, are important ways to understand and encapsulate scientific advancements and identify future research directions.

We can now conclude, after some reflection, that Amun was not wrong to be worried about the impact of exosomatic storage of information on his subjects. After all, as a powerful god, he might have been afraid that people would start using this information to develop self-consciousness.

## Author Contributions

GZ and AV contributed to the drafting and editing. Both authors contributed to the article and approved the submitted version.

## Conflict of Interest

The authors declare that the research was conducted in the absence of any commercial or financial relationships that could be construed as a potential conflict of interest.

## Footnotes

1 His work has been taken up in different directions, including in epistemology (how we know what we know; Bhaskar, 1993), theory of mind (especially non-dualistic psychology; Harré and Gillett, 1994), political philosophy (how political systems develop; Horkheimer and Adorno, 1947), and education and development (Vygotsky, 1987).
